# Modic changes in the lumbar vertebral column of chondrodystrophic and non-chondrodystrophic dogs with intervertebral disc disease

**DOI:** 10.3389/fvets.2024.1359016

**Published:** 2024-03-19

**Authors:** Dyah Agustini, Mary K. Heimann, Megan Co, Benjamin A. Walter, Devina Purmessur, Sarah A. Moore

**Affiliations:** ^1^Department of Veterinary Clinical Sciences, College of Veterinary Medicine, The Ohio State University, Columbus, OH, United States; ^2^Department of Biomedical Engineering, College of Engineering, The Ohio State University, Columbus, OH, United States

**Keywords:** Modic changes, intervertebral disc disease, lumbar, disc height index, MRI

## Abstract

**Introduction:**

Modic changes (MC) are signs of vertebral pathology visible on magnetic resonance (MR) images that have been associated with low back pain (LBP) and disc degeneration in people. Multiple breeds of dogs also develop MCs and coincident back pain. However, the association between breed, MC, and spinal pathologies has yet to be fully elucidated. This study aimed to identify the prevalence of MC that occur spontaneously in the lumbar vertebral column of dogs diagnosed with intervertebral disc disease (IVDD) and examine their association with demographic criteria and the disc width index (DWI).

**Methods:**

Medical records and lumbar vertebral column MR images were examined from 104 dogs (831 intervertebral disc spaces and adjacent vertebrae), which were divided into three groups: chondrodystrophic dogs (CD; *n* =54) and non-chondrodystrophic dogs (NCD; *n* =30) with IVDD as the primary diagnosis, and control dogs (*n* =20) with other spinal diseases as their primary diagnosis.

**Results:**

Increasing age and a diagnosis of IVDD were significantly associated with MC in dogs (*p* <  0.001 and *p* = 0.0062, respectively). In CD dogs with IVDD, Type 2 MC were most prevalent, whereas, in NCD dogs, Type 3 MC were the most prevalent type. Type 2 MC were distributed nearly equally across the lumbar vertebral column, while Type 3 MC were primarily detected at the level of L7-S1.

**Discussion:**

This study demonstrated that MC developed spontaneously in dogs, are common in dogs diagnosed with IVDD, and the type observed varies by breed. Further research is needed to understand the pathogenesis of MC; however, the increased presence of Type 2 MC in CD dogs, similar to what is found in people with disc degeneration, suggests that CD dogs could serve as models for MC in people.

## Introduction

Low back pain (LBP) is a worldwide leading cause of disability with a growing socioeconomic impact and rising incidence (1). Intervertebral disc disease (IVDD) is the most common cause of LBP (2, 3). In addition, both IVDD and LBP are some of the most common diseases of the vertebral column in both human and veterinary patients, particularly in dogs (4–6). Given the high prevalence of these conditions in clinical settings, a better understanding of the pathophysiology of these conditions and drivers of associated pain is needed to help develop new treatment strategies (3). Both people and dogs experience similar degenerative mechanisms that result in nucleus pulposus (NP) and anulus fibrosus (AF) structural alterations and associated IVDD (7). The clinical and biological similarities between people and dogs suggest that what is known about various contributors to LBP in people might have relevance to dogs and, conversely, that dogs with naturally occurring IVDD might serve as a spontaneous canine model for translational research (8).

**Figure 1:**
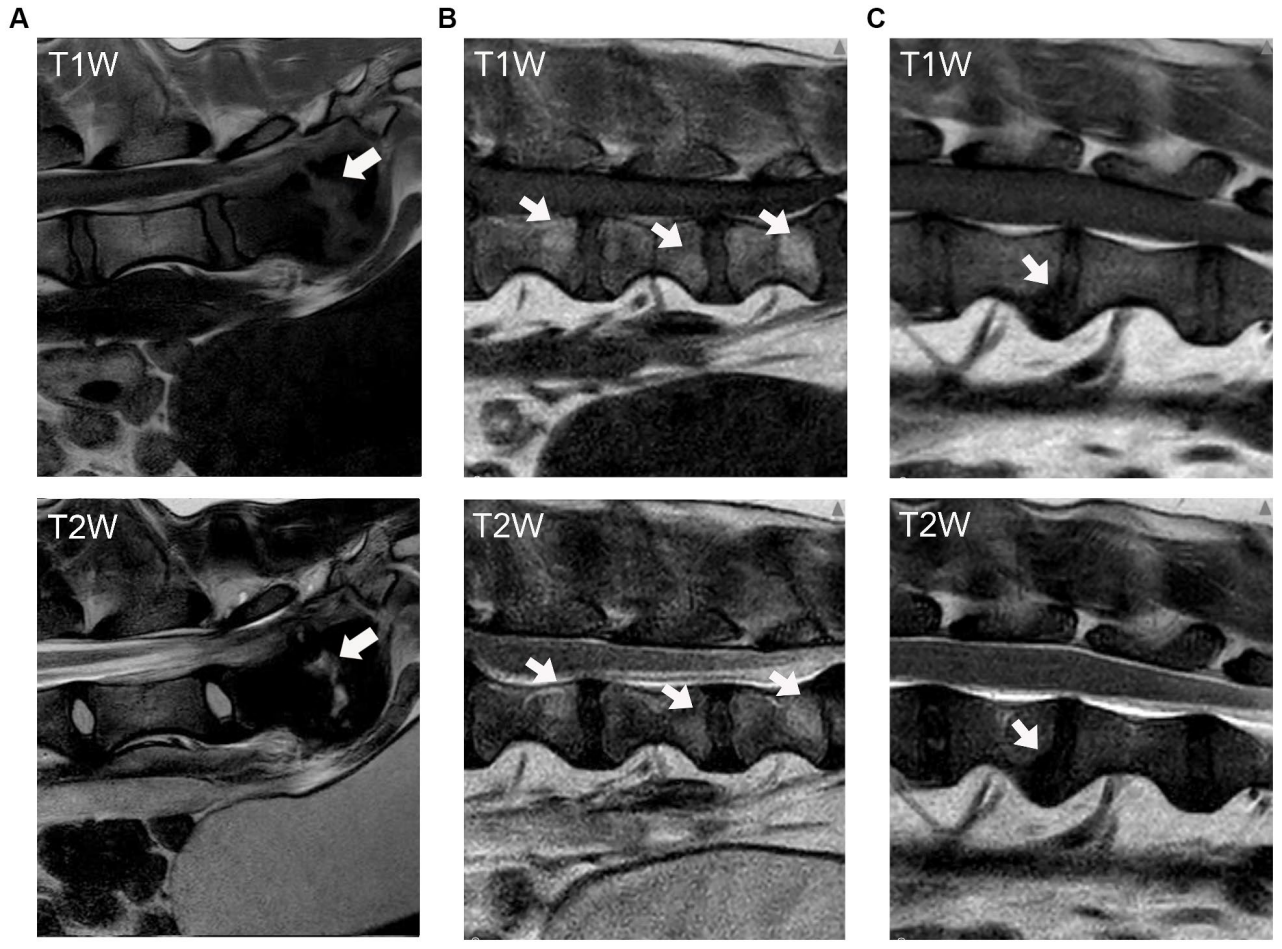
Modic changes as seen on T1-weighted and T2-weighted MR images of the canine vertebral column. The arrows indicate the signal changes in the vertebral body and endplate. **(A)** Type 1 MC, hypointensity on T1W and hyperintensity on T2W images. **(B)** Type 2 MC, hyperintensity on both T1W and T2W images; and **(C)** Type 3 MC, hypointensity on both T1W and T2W images.

Modic changes (MC) are pathologies of the vertebral column reflected as abnormalities of vertebral and cartilage endplates, leading to changes in signal intensity on magnetic resonance (MR) imaging studies (Figure 1). In people, MC are classically linked to LBP and IVDD (9). Three types of MC have been described, which are differentiated based on the type of signal changes observed: Type 1 MC are hypointense on a T1-weighted image and hyperintense on a T2-weighted image, Type 2 MC are hyperintense on both T1 and T2-weighted images, and Type 3 MC are hypointense on both T1 and T2-weighted images (10). The pathologic processes associated with the development of MC involve inflammation, fatty marrow infiltration, bone remodeling, and fibrosis (10, 11). These changes are correlated with back pain and putatively associated with chemical and mechanical stimulation of nerve fibers induced by the damaged vertebral body (12, 13). Disruption in the cartilage endplate and subchondral bone also contributes to loss of function and destabilization of the NP, which can lead to nerve compression and cause pain in dogs with IVDD (14).

Few studies have previously evaluated MC in dogs with spontaneous IVDD (15–17). The lumbosacral region has been previously noted as the most prevalent location (15, 17). The presence of hyperintense endplate lesions in T1-weighted and T2-weighted images of the thoracolumbar vertebral column has also been reported, indicating fatty infiltration of bone, a finding consistent with Type 2 MC (15). This finding was particularly prevalent in Dachshunds, raising the question of whether chondrodystrophic breeds might have a higher incidence of certain types of MC (15). Type 2 and Type 3 MC have also been previously reported as the most frequent endplate changes found in the canine vertebral column (16, 17). However, little is written about the influence of breed on types of MC observed in dogs (15–17). Therefore, further research is needed to understand better the etiology, prevalence, and distributions of MC in the canine vertebral column.

The goal of this study was to evaluate the prevalence and types of MC that occur spontaneously in the lumbar vertebral column of both chondrodystrophic (CD) and non-chondrodystrophic (NCD) client-owned dogs presented to a veterinary neurology referral center and ultimately diagnosed with IVDD. We also aimed to examine the association between the presence of MC and age, breed, body weight, sex, and the disc width index (DWI), which is also referred to as disc height index in humans and other animal models (18–20). Studying spontaneously occurring MC in dogs may provide novel insight into potential drivers of IVDD associated back pain and could provide a clinically relevant comparison with the human condition, providing support for future translational efforts.

## Materials and methods

This study retrospectively utilized medical records and MRI findings from client-owned dogs admitted to the OSU Veterinary Medical Center with MR imaging of the lumbar vertebral column performed for the clinical diagnosis of spinal disease.

### Data collection

#### Identification of cases

A search of the electronic medical records system of the Ohio State University Veterinary Medical Center was conducted to identify all dogs that had an MRI of the lumbar vertebral column between August 1, 2020, and September 15, 2022. The exclusion criteria were as follows:

1. Dogs had incomplete demographic data in their medical records, including age, body weight, sex, and breed,
2. The sagittal T1-weighted and T2-weighted MR images from T13-S1 were not available for review,
3. Dogs had a history of previous lumbar spinal surgery.

#### Definitions of groups

Dogs were divided into two main groups: IVDD and control groups. Those with a primary MRI diagnosis of IVD herniation, extrusion, or protrusion served as the IVDD group. Dogs with another primary diagnosis and no evidence of disc herniation, extrusion, or protrusion on their MRI served as the control group.

##### IVDD group

Dogs in the IVDD group were further divided into chondrodystrophic (CD) and non-chondrodystrophic (NCD) subgroups. Those identified in the medical record as mixed-breed dogs were excluded from the IVDD group due to the inability to characterize the CD status of their breed. Pure breed dogs included in the IVDD group were categorized as CD or non-CD dogs based on the published prevalence of their CFA12 and CFA18 *FGF4* retrogenes expression (21), and 0.10 or 10% frequency of CFA12 *FGF4* retrogene expressed was used as the threshold above which dogs were categorized as CD.

##### Control group

Dogs with a primary diagnosis other than IVDD (i.e., spinal neoplasia, neuritis, myelitis, etc.) and no secondary diagnosis of IVDD (defined as no evidence of disc protrusion, extrusion, or herniation) or dogs with no remarkable findings on their MRI report were included in the control group regardless of breed.

### MRI analysis for Modic changes

#### Image acquisition

Imaging for all dogs was originally performed by the Radiology Department of the Veterinary Medical Center at the Ohio State University using a Phillips Achieva 3.0 T MRI system (Highland Heights, Ohio 44,143) as part of standard care for diagnosis and management of their clinical complaint. A board-certified veterinary radiologist interpreted the imaging results and provided a final written report on the images. This report was retrieved from the medical record and used to categorize dogs as having a primary diagnosis of IVDD or other (control). Complete MRI studies for all dogs were retrieved and evaluated prospectively by three reviewers (DA, MC, MKH) to document the prevalence and type of MC present throughout the lumbar vertebral column.

#### Reviewer training

Reviewers underwent a standardized training protocol delivered by a board-certified veterinary neurologist (SM) to review MR images and identify MC. A written protocol for MC evaluation procedures was provided during the training as a step-by-step guide to image examination. Several example images of MC were provided in the protocol to help the reviewers easily identify their appearance. Five cases were initially selected as sample images for training purposes. After each reviewer evaluated the sample images individually, a group training session was conducted to compare interpretations, assess reviewer competence, and further harmonize the approach to review. The reviewers began the interpretation of the entire set of imaging studies only after competence was demonstrated.

### Image evaluation

#### Assessment of Modic changes

Reviewers individually assessed the presence (yes/no) and type of MC (1/2/3) present in each pair of vertebral endplates adjacent to the IVDs from T13-L1 to L7-S1 on the MR images. For vertebral levels where there was no perfect agreement between all three reviewers, an additional assessment by a board-certified veterinary neurologist determined the final judgment.

Imaging studies were evaluated in a randomized fashion using a RocketPACS web-based system (VetRocket, Santa Clara, CA), and reviewers were blinded to the diagnosis and group allocation of each dog. Reviewers were instructed to evaluate the appearance of the cranial and caudal vertebral endplates adjacent to the IVDs from T13-S1 on both T1-weighted and T2-weighted sagittal images to determine the presence and type of MC observed (Figure 1). Definitions of MC applied in this study were taken from Modic et al. (10):

1. Type 1 MC (hypointensity on T1W and hyperintensity on T2W images),
2. Type 2 MC (hyperintensity on T1W and hyperintensity on T2W images),
3. Type 3 MC (hypointensity on both T1W and T2W images).

#### Disc width index measurement

**Figure 2:**
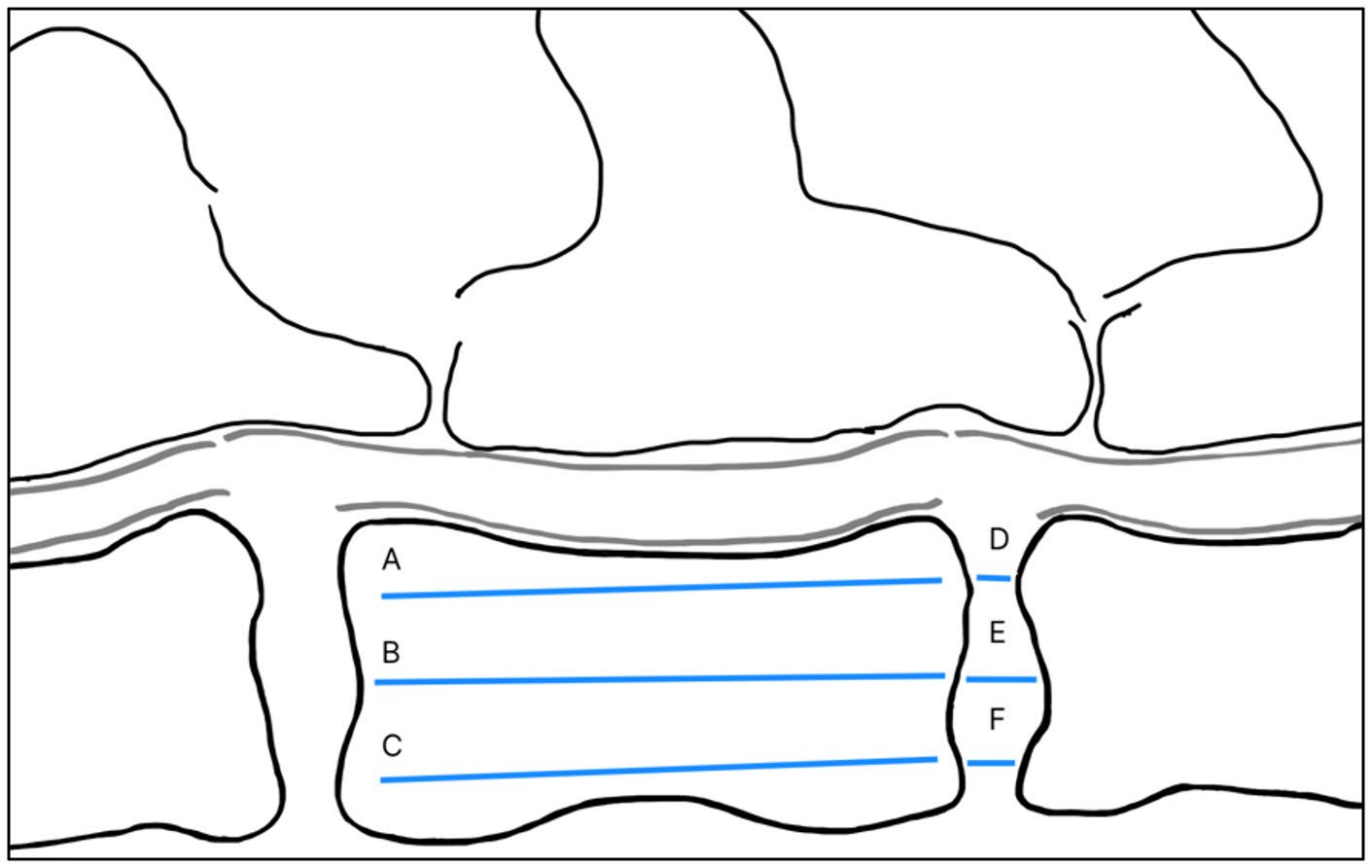
Illustration of DWI measurement. A and D refer to the dorsal side of a vertebral body and intervertebral disc, whereas C and F refer to the ventral side. A, B, and C refer to the vertebral body cranial to the adjacent disc. Image and measurement were adapted from Lü et al. (20).

A mid-sagittal T1W image was used to calculate the DWI for each individual disc width as a potential marker of disc degeneration. The calculation for DWI was based on the ratio of the total caudal-cranial length of the intervertebral disc relative to the vertebral body (20):


$$DWI=\frac{\left(D+E+F\right)}{\left(A+B+C\right)}\text{,}$$

where D, E, and F are the measurements of intervertebral disc width, and A, B, and C are the measurements of the vertebral body (Figure 2). A single reviewer (DA) performed DWI measurements. Five cases were chosen at random from the clinical data set and each case was assessed three different times to evaluate Intra Class Correlation (ICC) analysis to ensure test–retest reliability.

### Statistical analysis

Statistical analysis was conducted using STATA/BE 17.0 statistical software. An intra-class correlation test was performed to assess the intra-rater reliability of DWI measurement and inter-rater reliability of MC assessment during reviewer training. Dog breed, age, sex, and body weight were reported using descriptive statistics. Comparison of the number and type of MC present between groups was assessed using cross-tabulation analysis. A logistic regression test assessed the association between MC and age, body weight, sex, and DWI as predictors. Fisher’s Exact test was used to test each group’s independence (control, CD, and NCD dogs) with the occurrence of MC. The mean differences between DWI of vertebrae with MC and without MC was examined using a two-sample *t*-test. Statistical differences were considered significant if the *p*-value was <0.05.

## Results

### Case selection

**Figure 3:**
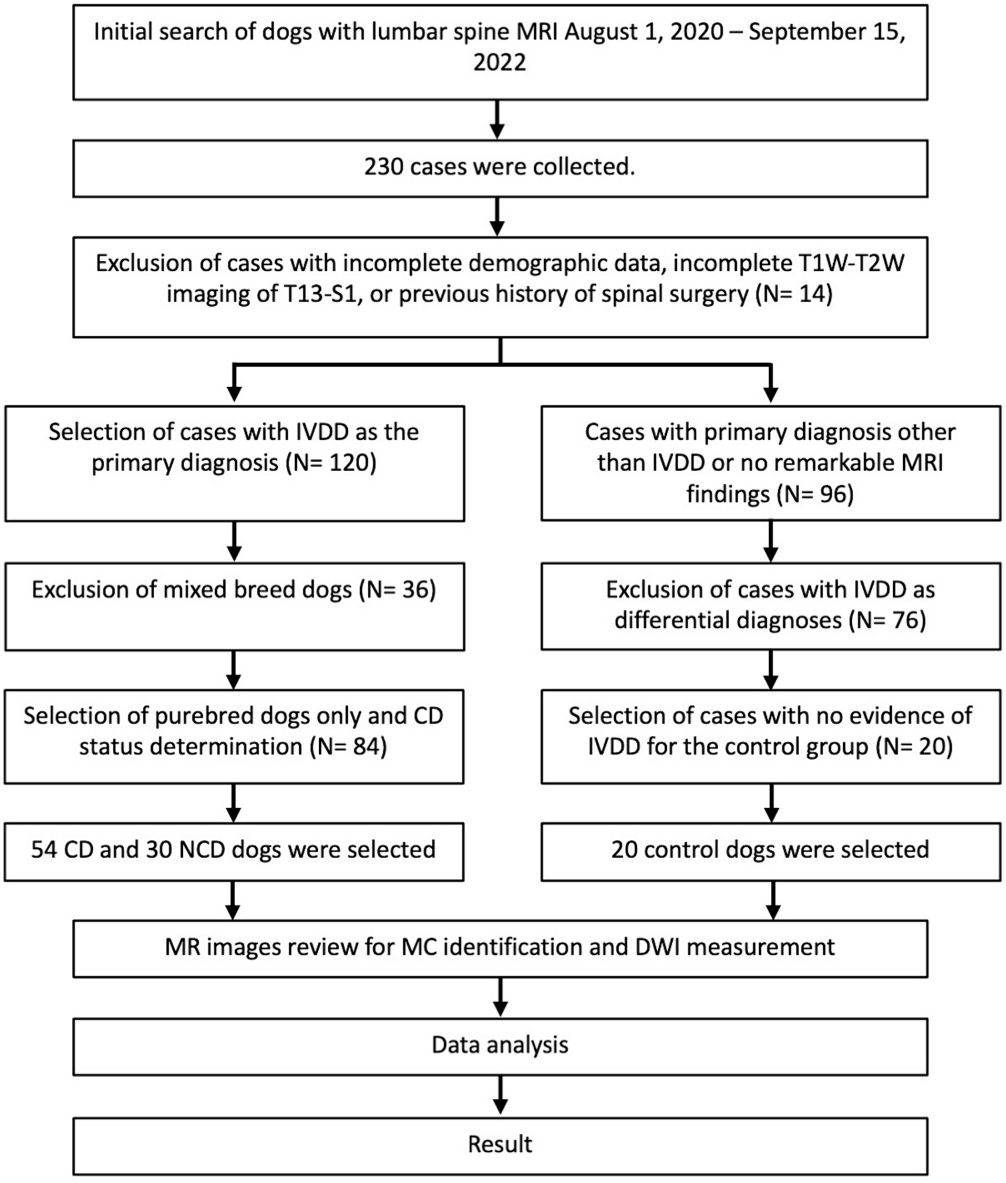
Flow chart of the case selection process for 104 dogs with and without intervertebral disc disease.

Two hundred thirty dogs with lumbar vertebral column MR images were identified through the initial medical records search. Cases with incomplete demographic information, incomplete MRI studies, or a history of previous spinal surgery were excluded (*n* = 14). Cases with IVDD as the primary diagnosis (*n* = 120) were separated from those with other primary MRI diagnoses or normal MRI (*n* = 96). Only pure-breed dogs were included in the IVDD group (*n* = 84), further divided into CD and NCD groups (*n* = 54 and *n* = 30, respectively). From the group of dogs with normal MR images (no remarkable finding) or other primary diagnoses, dogs with a secondary diagnosis of intervertebral disc herniation, protrusion, or extrusion in their MRI result were excluded (*n* = 76) to obtain a group of dogs with no MRI indication of IVDD that were used as a control group (*n* = 20). Vertebrae with fractures involving the endplate or where the entire disc and endplate were not visible in the sagittal image due to truncation were removed from the image review process (*n* = 4). Two dogs from the IVDD groups and one from the control group had eight lumbar vertebrae, resulting in additional vertebrae for evaluation. In total, 831 intervertebral discs and their adjacent vertebral endplates were evaluated from all three groups (Figure 3).

### Demographics of the cohort

MRI studies from a total of 104 dogs were evaluated (54 CD dogs, 30 NCD dogs, and 20 control dogs). There was no statistically significant difference in sex distribution between the groups (*p* = 0.154), whereas age and body weight were significantly different (*p* < 0.001 for both). The demographic features of each group are summarized in Table 1.

**Table 1:** Demographics of 104 client-owned dogs with and without IVDD.

Demographic criteria	Control group (*n* = 20)	CD group (*n* = 54)	Non-CD group (*n* = 30)	*p-*value
Age (year)				<0.001*
Median	3	7	8	
Range	1–9	2–13	2–12	
Body weight (kg)				<0.001*
Median	20.4	8.2	36.8	
Range	6.5–57	3–34.6	7.8–69.4	
Sex				
Male-neutered	6	33	22	0.154**
Male intact	4	1	1	
Female-spayed	9	20	7	
Female intact	1	0	0	

*Kruskal-Wallis test. **Fisher’s exact test.

The control group mainly consisted of mixed breed dogs (*n* = 6), Border Collie (*n* = 4), Australian Shepherd (*n* = 3), and German Shepherd (*n* = 2). Breeds such as American Pitbull Terrier, Belgian Malinois, English Setter, French Bulldog, and German Shorthaired Pointer represented only one of each breed. The most represented breeds in the CD group were Dachshund (*n* = 30), Beagle (*n* = 7), French Bulldog (*n* = 6), and Bichon Frise (*n* = 3). Other breeds included in lesser numbers (*n* ≤ 2) in the CD group were American Cocker Spaniel, Basset Hound, Jack Russell Terrier, Pembroke Welsh Corgi, Poodle, and Shih Tzu. The most represented breeds in the NCD group were German Shepherd (*n* = 10), Great Dane (*n* = 3), and Labrador Retriever (*n* = 3). The NCD group also contained one each of the following: Australian Shepherd, Belgian Malinois, Bernese Mountain, Border Collie, English Bulldog, English Coon Hound, Eskimo, Fox Terrier, German Shorthaired Pointer, Golden Retriever, Greyhound, Pomeranian, Shetland Sheepdog, and Shilo Shepherd.

### Modic changes in canine lumbar vertebral column

**Figure 4:**
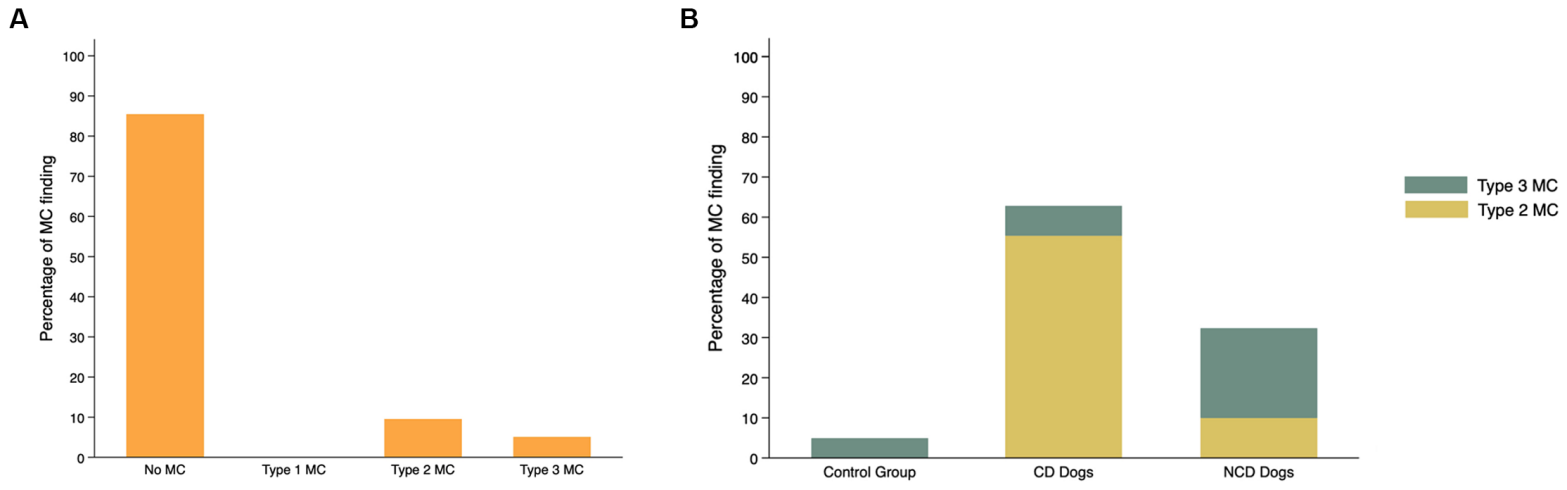
Frequency (%) of Modic changes (MC) observed in 831 lumbar vertebral segments from 104 dogs with and without intervertebral disc disease (IVDD) **(A)** by type of MC observed and **(B)** by group and breed chondrodystrophy status.

**Figure 5:**
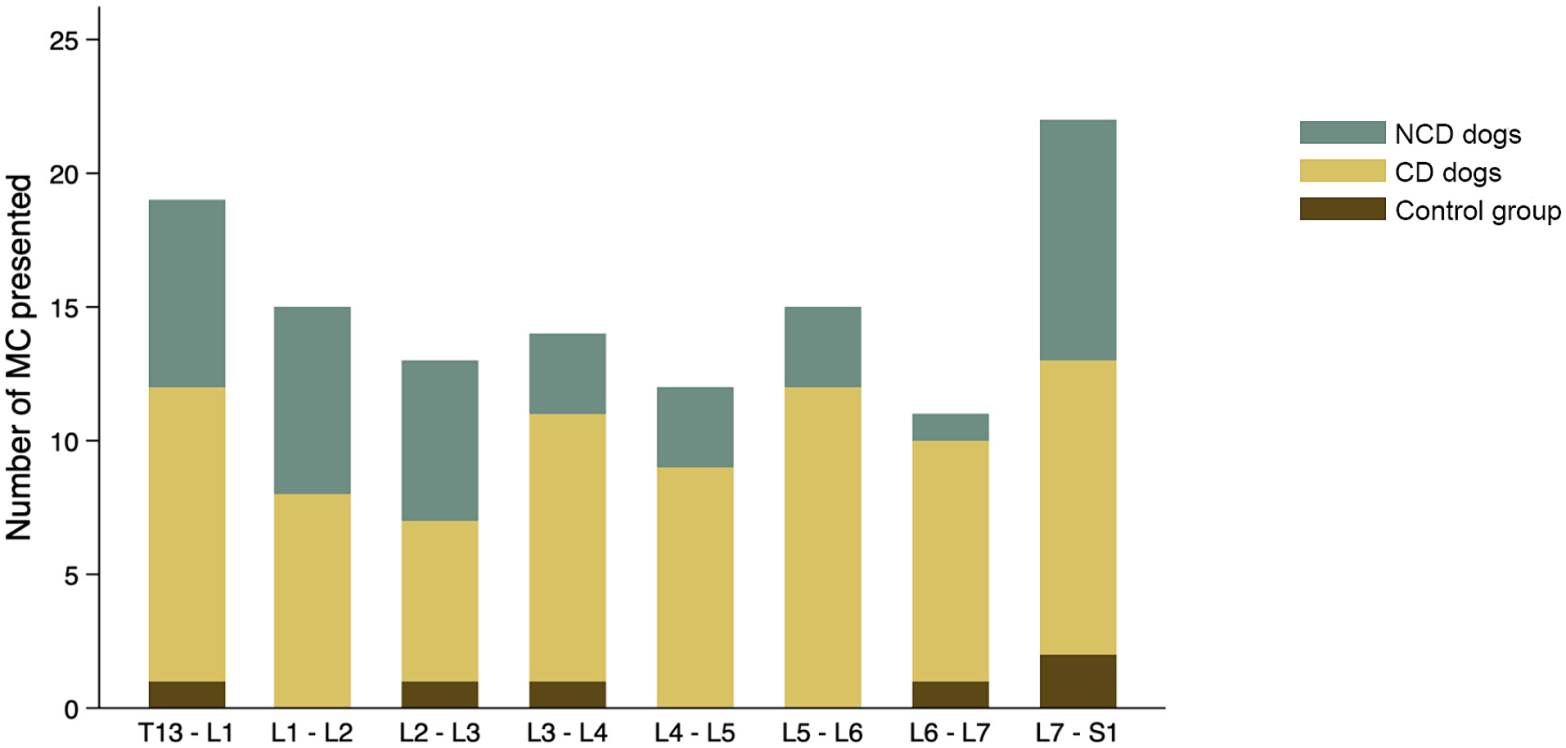
Distribution of all Modic changes observed in 831 lumbar vertebral segments from 104 dogs with and without intervertebral disc disease (IVDD). Dogs were divided into a control group with no evidence of IVDD, a group of chondrodystrophic dogs (CD) with IVDD and a group of non-chondrodystrophic dogs (NCD) with IVDD.

The findings with respect to the presence and type of MC observed are summarized in Table 2. Of all lumbar vertebral levels evaluated, 85.4% showed no MC, 9.5% showed Type 2 MC, and 5.1% showed Type 3 MC. No Type 1 MC changes were observed in any dog included in the present study (Figure 4A). Type 2 MC were found to be presented in CD and NCD dogs but not in control dogs, while Type 3 MC were detected in all dogs, including the control group (Figure 4B). In the IVDD groups, two CD dogs and one NCD dog were found to have both Type 2 MC and Type 3 MC within their lumbar vertebral column, which we refer to as presenting a mixed type of MC (Table 2). In general, Type 2 MC were distributed widely throughout the lumbar vertebral column, whereas Type 3 MC were detected mostly in vertebral endplates adjacent to the L7-S1 disc space (*n* = 13) (Figure 5).

**Table 2:** Comparison of Modic changes identified in 104 chondrodystrophic (CD) and non-chondrodystrophic (NCD) dogs with and without IVDD.

Modic changes	Control group (*n* = 20)	CD group (*n* = 54)	Non-CD group (*n* = 30)	*p*-value (Fisher’s exact test)
No MC found	17 (85%)	25 (46.3%)	15 (50%)	< 0.001
Type 1 MC	0	0	0	
Type 2 MC	0	21 (38.9%)	3 (10%)	
Type 3 MC	3 (15%)	6 (11.1%)	11 (36.7%)	
Mixed type (2/3)	0	2 (3.7%)	1 (3.3%)	

**Figure 6:**
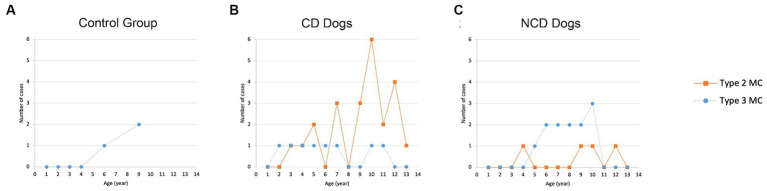
Distribution of Modic changes based on age in **(A)** control group, **(B)** CD dogs, and **(C)** NCD dogs.

The presence of MC was not significantly associated with sex (*p* = 0.264) or body weight (*p* = 0.263). Age appeared to be significantly associated with MC (*p* < 0.001). The distribution of MC based on age for all groups are presented in Figure 6. The odds ratio (OR) of having MC in the lumbar vertebral column increased by 1.36 times (95% CIs = 1.174–1.575) for each one-year increase in age. The frequency of MC was higher in by CD and NCD dogs with IVDD compared to controls dogs (*p* = 0.0062), where the odds ratio for the presence of MC in CD dogs was 6.57 (95% CIs = 1.723–25.076) and in the NCD dogs was 5.67 (95% CIs = 1.369–23.462).

**Figure 7:**
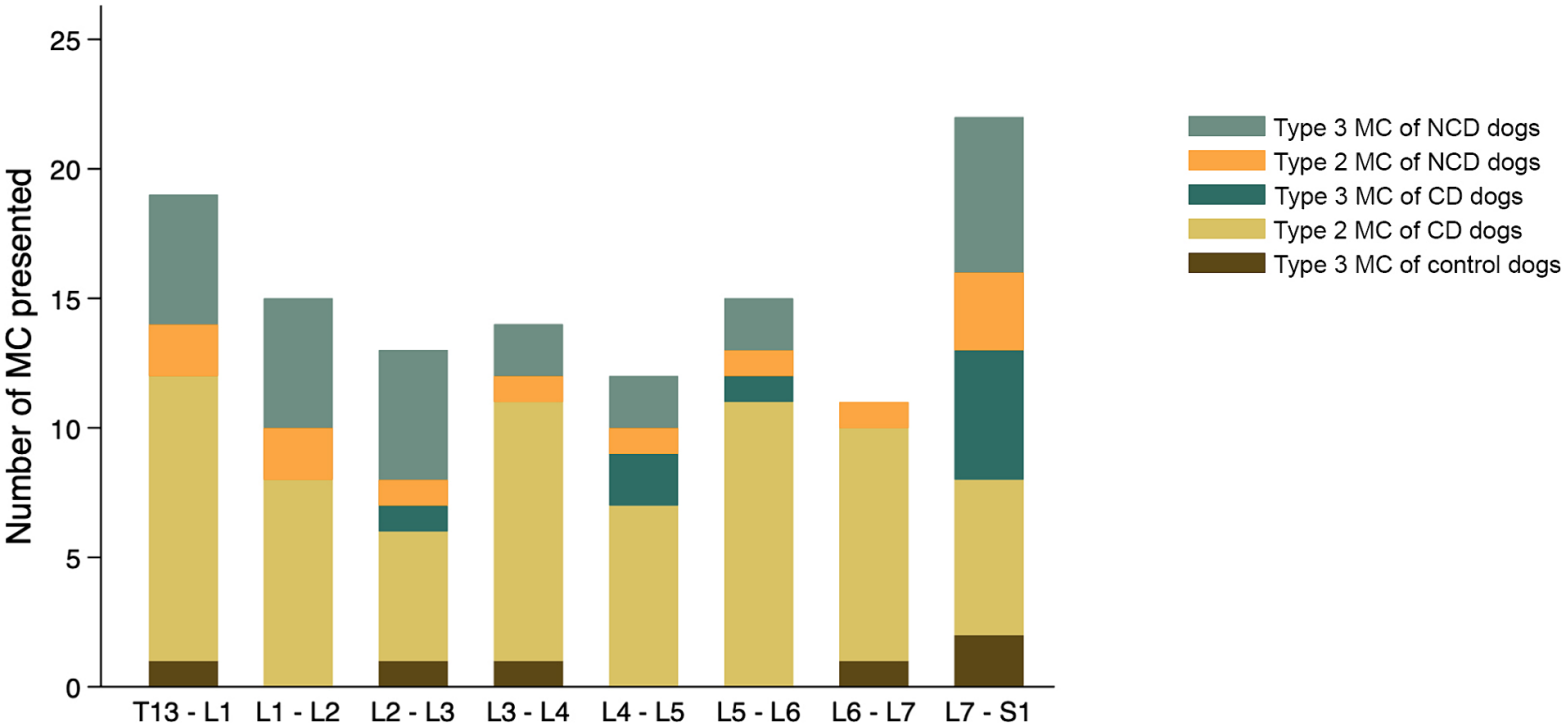
Distribution of Modic changes, by type, observed in 831 lumbar vertebral segments from 104 dogs with and without intervertebral disc disease (IVDD). Dogs were divided into a control group with no evidence of IVDD, a group of chondrodystrophic dogs (CD) with IVDD and a group of non-chondrodystrophic dogs (NCD) with IVDD.

The distribution of each MC type and its location by vertebral level are summarized in Figure 7. In the control group, two of six Type 3 MC were found in vertebral endplates adjacent to the T13-L1 intervertebral disc, while the remainder were located at the level of L7-S1. Type 2 MC were more commonly observed than Type 3 MC in the CD dogs (*n* = 67 and *n* = 9, respectively), whereas Type 3 MC were observed more than Type 2 MC in the NCD dogs (*n* = 27 and *n* = 12, respectively). In both CD and NCD dogs, Type 2 MC were distributed throughout the lumbar vertebral column, while Type 3 MC occurred predominantly at the vertebral endplates adjacent to the L7-S1 intervertebral disc space.

### Relationship between Modic changes and disc width index

The intraclass-correlation test (ICC) was performed under a two-way mixed-effects model to assess the repeatability of a single observer’s measurements of disc width in dogs with and without IVDD as measured at three distinct times. Intra-observer reliability for DWI measurements was 0.99, which can be categorized as excellent (22).

The average ratio of intervertebral disc width was 0.25–0.4 for the disc space adjacent to vertebrae with MC and 0.26–0.35 for the disc space adjacent to vertebrae without MC present. There was no statistically significant difference in DWI between disc adjacent to vertebral endplates that contained MC and those that did not, except at the level of L7-S1, where the DWI was significantly larger in discs adjacent to vertebral endplates with MC (*p* = 0.0247) (Table 3).

**Table 3:** Comparison of mean DWI in each intervertebral disc adjacent to vertebral endplates with and without MC.

Level	Mean disc width index		Two-sample *t*-test (*p*-value)
	Normal (SD)	With MC (SD)	
T13-L1	0.26 (0.05)	0.25 (0.05)	0.8726
L1-L2	0.24 (0.05)	0.25 (0.05)	0.6147
L2-L3	0.26 (0.2)	0.26 (0.05)	0.9521
L3-L4	0.22 (0.05)	0.234 (0.04)	0.4178
L4-L5	0.23 (0.2)	0.21 (0.05)	0.7742
L5-L6	0.22 (0.2)	0.214 (0.04)	0.9497
L6-L7	0.23 (0.05)	0.24 (0.05)	0.5851
L7-S1	0.35 (0.08)	0.4 (0.2)	0.0247

## Discussion

This study represents one of only a few publications evaluating MC in dogs (15–17, 23) and is the first to associate the type and distribution of MC with breed chondrodystrophy status in client-owned dogs. Findings from our study support those recently published by Beukers et al. (17) that dogs can spontaneously develop MC, that MC are commonly observed at the lumbosacral junction, and that the development of MC in dogs is strongly associated with age, a finding observed in people as well.

In our study population, no Type 1 MC were identified and Type 2 MC were the most common type of MC observed. The Type 1 MC images presented in Figure 1A were obtained from another client-owned dog diagnosed with discospondylitis and not included in the current study. It is interesting to note that Beukers et al. (17) also found that Type 1 MC was significantly associated with imaging characteristics of discospondylitis. Type 1 MC are thought to reflect the replacement of normal bone marrow by fibrous and granulation tissue. TNF-α expression in the vertebral endplates of people with Type 1 MC is significantly higher than in those with Type 2 MC (24), supporting the argument that Type 1 MC are most likely to be associated with active inflammation (9). Type 1 MC have also been significantly associated with the presence of low back pain, and disc herniation appears to be an important risk factor for development (25, 26). Type 1 MC are also considered an interconvertible lesion, as they can develop into Type 2 MC or revert to normal condition (19, 26–28). Given what is known about the underlying pathophysiology and time course for the development of Type 1 MC, we assume it is possible that Type 1 MC were not observed in our population of dogs because they represent very early degenerative changes resulting in more mild clinical signs that could be missed by owners and veterinarians until the process becomes more advanced. In the previous studies, Type 1 MC accounted for a minimal number of dogs (16, 17). The presence of Type 1 MC has also potentially been associated with previous spinal surgery (17). Our study design excluded dogs with previous spinal surgery from evaluation and therefore precluded assessing this relationship. Additionally, it is possible that Type I MC represent an earlier stage of vertebral endplate changes associated with IVDD (11, 15) and that Type I MC can transition to Type 2 MC over time (10, 19, 26, 28); therefore, more subtle signs of lower back pain associated with Type I MC could be missed in dogs who are unable to self-report their back pain.

Another recent study evaluating MC in the canine vertebral column observed Type 3 MC most commonly (17); however, several studies have shown that Type 2 MC are the most commonly observed type of MC in people and are associated with the replacement of normal bone marrow with fat and upregulation of complement-mediated inflammation. Type 3 MC, which represent subchondral bone sclerosis, are reportedly more rare in people (19, 29, 30). An important difference in population between Beukers et al. (17) and the present might drive observed differences in results, specifically the inclusion of a large number of CD dogs in the present study. In our canine IVDD population, 65.4% of MC detected were Type 2 MC, while 34.6% of MC identified were Type 3 MC, suggesting that both types of MC occur relatively frequently in dogs with IVDD. Type 2 MC were detected most frequently in CD dogs, while only 10% of NCD dogs in our cohort had Type 2 MC. In contrast, Type 3 MC were observed more commonly in NCD dogs.

While not an aim of their study, another publication describing MRI findings associated with IVDD in a population composed primarily of CD dogs also reported a high prevalence of Type 2 MC (16), whereas Type 3 MC were most common in a previously reported cohort predominantly composed of NCD (17). Given the increased commonality of Type 2 MC in both people and CD dogs, our results could support the CD dog as a more translationally relevant spontaneous canine model for studying MC as compared to the NCD.

Across all dogs in the current study, MC were observed most frequently in the vertebral endplates at the level of L7-S1, and this finding is similar to previous reports (15, 17). However, we observed distinct patterns of distribution for Type 2 MC and Type 3 MC. Type 2 MC were observed mostly in CD dogs and were distributed almost equally across all lumbar segments, whereas Type 3 MC were observed mostly in NCD dogs and predominated at the vertebrae adjacent to the L7-S1 intervertebral disc space. To our knowledge, our study is the first to report a difference in MC type and distribution between CD and NCD dogs, and these findings might be important in considering causes and clinical implications of MC. We speculate these distinct distribution patterns might be influenced by differences in genetics, pathophysiology or biomechanics underlying IVDD characteristics in CD and NCD dogs. In the CD dogs, the CFA12 *FGF4* retrogene is considered a major risk factor for the IVDD phenotype. Histologically, discs from CD dogs undergo loss of notochordal cells and transformation of nucleus pulposus into chondrocyte-like cells at a young age (21, 31). This predisposes to early disc degeneration, which can occur in a similar manner throughout the vertebral column (32–34). Conversely, in NCD dogs, risk factors for the development of IVDD appear to center more on age, body dimensions, and vertebral column biomechanics (33, 35, 36), where relative ventrodorsal instability make the lumbosacral junction focally and particularly susceptible to high wear and tear and associated age-related disc degeneration (32, 33, 35).

Several studies in people report that vertebral endplates adjacent to the lower lumbar IVDs, L4-L5 and L5-S1, are the most common sites for Type 2 MC (13, 29, 37). This differs from the findings in our cohort of dogs. The differences might be caused by the distinction in biomechanical factors affecting the vertebral column between dogs and people or could be driven by the unique role of *FGF4* retrogene expression as a driver of IVDD in CD dogs (21, 31, 38). Comparably, a mutation in the fibroblast growth factor receptor 3 (*FGFR3*) in people, which causes an achondroplasia phenotype (39) is associated with lumbar spinal stenosis and more diffuse degenerative changes throughout the entire lumbar vertebral column, differing from the high incidence of IVDD focally at the lumbosacral junction in people without an achondroplasia phenotype (40, 41). More studies are needed to investigate the relationship between *FGF4* retrogene expression and vertebral endplate changes consistent with MC.

Imaging assessment of disc space narrowing through calculation of disc height index (analogous to DWI measured in the present study) is often used as a clinical indicator of IVDD in people (42) since intervertebral disc height is related to age-related degenerative changes (43, 44). A study conducted by Akeda et al. (42) showed that lumbar disc height reduction in elderly people was significantly associated with low back pain conditions. Changes in laminar layers of AF and deposition of chondroid substance that was found particularly in middle-aged and older people have been proposed to contribute to disc space narrowing (43, 45). The reduction of disc height index has also been significantly associated with the presence of MC in people, particularly Type 2 MC (19). In the present study, we did not observe an association between reduced DWI and the presence of MC. Instead, we found an increased ratio of DWI in dogs affected with MC, but only at the level L7 – S1. The lack of association between DWI and MC in our population might be related to the diverse breeds included in our study, leading to variability in vertebral body and disc width measurement. Previous research in the canine cervical vertebral column found that disc to vertebral body area and length ratios differed significantly by breed (46). Further morphometric studies of the canine vertebral column are needed to obtain more information about disc space narrowing association with IVDD and MC in dogs.

The Pfirmann grading system has been used as a tool to assess disc degeneration based on sagittal T2-weighted images in both people and dogs (47–49). A previous study investigating lumbar IVDD in people found that MC were correlated with higher Pfirmann grade (48). The present study did not employ Pfirmann grading as a marker of disc degeneration, as it is not commonly used as part of the clinical image evaluation process for dogs with IVDD. Future studies could prospectively explore the relationship between Pfirrman grade and the presence of MC in dogs with IVDD.

This study represents the first evaluation of the relationship between chondrodystrophy breed status and the presence and distribution of spontaneously developing MC. Our study leveraged a comprehensive set of clinically annotated MRI studies, coupled with dog demographic information, to evaluate associations between these factors and the type of MC developed in a veterinary patient population with potential translational relevance. Given the nature of our population, we were unable to correlate MRI observations with histopathologic findings and were unable to define a control group of dogs with entirely normal vertebral columns for comparison. Additionally, because MR images were obtained from a database of previously imaged veterinary patients, correlation with other parameters, such as semi-quantitative measures of back pain or owner-derived quality of life scores, were not possible in the present study. While this study focused on the presentation of MC in the lumbar region of canine vertebral column, evaluation of MC in the thoracic and cervical region may provide further insight into the MC prevalence and breed-associated distribution in dogs. To address these limitations, prospective longitudinal investigation in dogs, including imaging of the entire vertebral column, will be necessary to better understand the relationship between MC development, degenerative changes to the intervertebral disc, and their association with low back pain.

## Conclusion

This study demonstrates that MC occur spontaneously in dogs diagnosed with IVDD of the lumbar vertebral column. Age and diagnosis of IVDD are risk factors for the presence of MC, and older dogs and both CD and NCD dogs with IVDD have an increased odds ratio for MC. Type of MC observed varies with breed where 38.9% of CD dogs in our study population had Type 2 MC, which were distributed almost evenly throughout the lumbar vertebral column, while 36.7% of NCD dogs showed Type 3 MC, which were found mostly in the L7-S1 region. Further research is needed to understand the pathogenesis of MC and their correlation with clinical signs of back pain; however, the increased presence of Type 2 MCs in CD dogs, similar to what is found in people with disc degeneration, suggests that CD dogs might serve as a model for future translation studies of MC.

## Data availability statement

The raw data supporting the conclusions of this article will be made available by the authors, without undue reservation.

## Author contributions

DA: Conceptualization, Data curation, Formal analysis, Investigation, Methodology, Visualization, Writing – original draft, Writing – review & editing. MH: Investigation, Methodology, Writing – review & editing. MC: Investigation, Methodology, Writing – review & editing. BW: Conceptualization, Formal analysis, Methodology, Supervision, Writing – review & editing. DP: Conceptualization, Formal analysis, Methodology, Supervision, Writing – review & editing. SM: Conceptualization, Formal analysis, Investigation, Methodology, Supervision, Writing – review & editing.
